# Neonatal and maternal serum creatinine levels during the early postnatal period in preterm and term infants

**DOI:** 10.1371/journal.pone.0196721

**Published:** 2018-05-24

**Authors:** Hayato Go, Nobuo Momoi, Nozomi Kashiwabara, Kentaro Haneda, Mina Chishiki, Takashi Imamura, Maki Sato, Aya Goto, Yukihiko Kawasaki, Mitsuaki Hosoya

**Affiliations:** 1 Department of Pediatrics, Fukushima Medical University School of Medicine, Fukushima, Japan; 2 Center for Integrated Science and Humanities, Fukushima Medical University, Fukushima, Japan; The University of Manchester, UNITED KINGDOM

## Abstract

We investigated the relationship of neonatal and maternal serum creatinine (nSCr and mSCr, respectively) with various maternal/infant characteristics at different gestational ages (GA). We reviewed medical records of neonates admitted to NICU. We collected data on birth weight, GA, Apgar scores, medications, etc. Spearman’s test was used to analyze the correlation between serum creatinine and continuous variables, and the Mann-Whitney U and Kruskal-Wallis tests for continuous variables between groups. The changes in nSCr, mSCr, and nSCr/mSCr ratio because of gestational age and the points in gestational changes in trends were estimated using joinpoint trend analysis. From 614 neonate and mother pairs, we found that nSCr was significantly correlated with GA. However, mSCr at >28 wks decreased with GA. The nSCr/mSCr ratio was correlated with GA. In infants born <29 weeks, pregnancy-induced hypertension (PIH) (p = 0.000, β = 0.20) and mSCr (p = 0.000, β = 0.73) were significantly associated with nSCr. In term infants, maternal magnesium administration (p = 0.000, β = 0.25), respiratory distress syndrome (p = 0.013, β = 0.16), PIH (p = 0.005, β = 0.19), and mSCr (p = 0.000, β = 0.33) were significantly associated with nSCr. nSCr reflected mSCr at all gestational ages. The correlation between nSCr and mSCr in preterm infants (p = 0.000, β = 0.74) was stronger than in term infants (p = 0.000, β = 0.34).

## Introduction

Renal function is typically estimated by glomerular filtration rate (eGFR) [1]. The simplest and most commonly used parameter for eGFR measurement is serum creatinine (SCr) [2]. Lao et al. showed that neonatal serum creatinine levels (nSCr) correlated with birth weight (BW) and maternal serum creatinine (mSCr) [3]. Renal function is low at birth, especially in premature infants [4]. In addition, preterm birth is associated with maternal complications. Thus, nSCr in preterm infants is influenced by various factors [5,6,7]. While nSCr can be a useful surrogate marker of neonatal renal function, some perinatal complications such as pregnancy-induced hypertension (PIH), small for gestational age (SGA) may also affect fetal glomerular development by inducing intrauterine growth retardation [8,9,10]. However, there are few studies that have reported the relationship among nSCr, perinatal complications (PIH, SGA, etc.), and mSCr in all gestational ages in a large cohort. The objectives of this study were to investigate postpartum nSCr in infants delivered at different gestational ages and to study the relationship between nSCr and mSCr in a representative population sample of preterm infants.

## Materials and methods

### Study population

This retrospective cohort study took place in the neonatal intensive care unit (NICU) of Fukushima Medical University Hospital, Japan between 2006 and 2014. Recruitment of neonates for this study was limited to neonates admitted to this NICU. The study was approved by the ethics committee of Fukushima Medical University. The additional inclusion criteria was daily urine output > 1.0 ml/kg/h. Exclusion criteria included congenital anomalies, oligohydramnios, and severe renal malformations diagnosed by prenatal ultrasound. Preterm infants were recruited and followed until discharge.

### Creatinine measurement

Blood samples were collected through a cord blood or peripheral venipuncture for serum creatinine measurements from each neonates at birth, day 14, and one month and plasma creatinine day 7. Although we routinely measured serum tests at birth, day 14 and day 28 for other research, we collected plasma on day 7 to save the volume. And blood samples of the mother were collected before birth. Creatinine was measured using a sarcosine oxidase enzymatic assay on TBA-c16000 (Toshiba, Japan). The method of analysis remained unchanged during the study period.

### Prenatal risk factors and postnatal risk factors

We determined PIH, hemolytic low platelet syndrome (HELLP) syndrome, antibiotic administration, steroid administration, and magnesium administration, as prenatal risk factors, and GA, BW, gender, indomethacin administration, neonatal antibiotic administration, SGA (birth weight <10^th^ percentile), and Apgar score as postnatal risk factors.

### General management of infants

In case of hypotension, neonates were treated with an inotropic or volume expander and hydrocortisone. The infants were cared for in heated and humidified incubators. The incubator was controlled to an abdominal skin temperature of 36.5 °C– 37.5 °C, with a relative humidity of 90% especially in preterm infants. Initially, the neonates were given intravenous fluids at 70–90 mL/kg/day during the first 24h of life, which was progressively increased to 100 mL/kg/day by 48–72 h of age.

The amount of intravenous fluids administered was reduced when infants achieved 100 mL/kg/day of enteral intake; the feeds were progressively advanced, thereafter, to a volume of 140–160 mL/kg/day. Parenteral nutrition was commenced as soon as possible (generally by 8–36 h postpartum) in premature neonates born at < 30 wks. Blood pressure was monitored by continuous invasive blood pressure monitoring for premature neonates born at <27 wks, whenever possible. While prophylactic indomethacin was not used, we administered indomethacin to infants with hemodynamically significant patent ductus arteriosus.

Most of the infants born at < 28 wks were ventilated using conventional ventilation or high-frequency oscillatory ventilation. Infants with respiratory distress syndrome (RDS) received intratracheal surfactant.

### Statistical analysis

Data on antenatal problems, use of antenatal steroids, maternal magnesium administration, PIH, HELLP, BW, GA, gender, Apgar score, SGA, RDS, mSCr and nSCr were collected from medical records. Continuous variables were presented as medians. Data were analyzed using SPSS for Windows, release 11.0 (SPSS, Chicago, IL, USA). Correlation between the variables of BW, GA, Apgar Score, mSCr, and nSCr were analyzed using Spearman’s rank correlation (r). For continuous variables, the Mann-Whitney U and Kruskal-Wallis tests were used for continuous variables between groups. Variables at a p level < .05 at univariate analysis were entered into a multiple regression analysis. Standardized coefficient was expressed as β. Multicollinearity of independent variables was assessed by using variance inflation factor (VIF). VIF exceeding is regarded as indicating serious multicollinearity, and values grater than 4.0 may be a cause for concern. Due to high correlation between BW and GA, each variable was entered separately into Models 1 and 2. BW was entered into Model 1, and GA was entered into Model 2.

The changes in nSCr, mSCr and nSCr/mSCr ratio due to gestational age and the points in gestational changes in trends were estimated using joinpoint trend analysis. Recently, this method has been applied to not only birth trends and cancer trends, but to clinical examination values as well [11]. We used the Joinpoint Regression Program ver. 3.5 (Statistical Methodology and Applications Branch and Data Modeling Branch, Surveillance Research Program, National Cancer Institute). A value of p < 0.05 was considered statistically significant.

## Results

**Fig 1:**
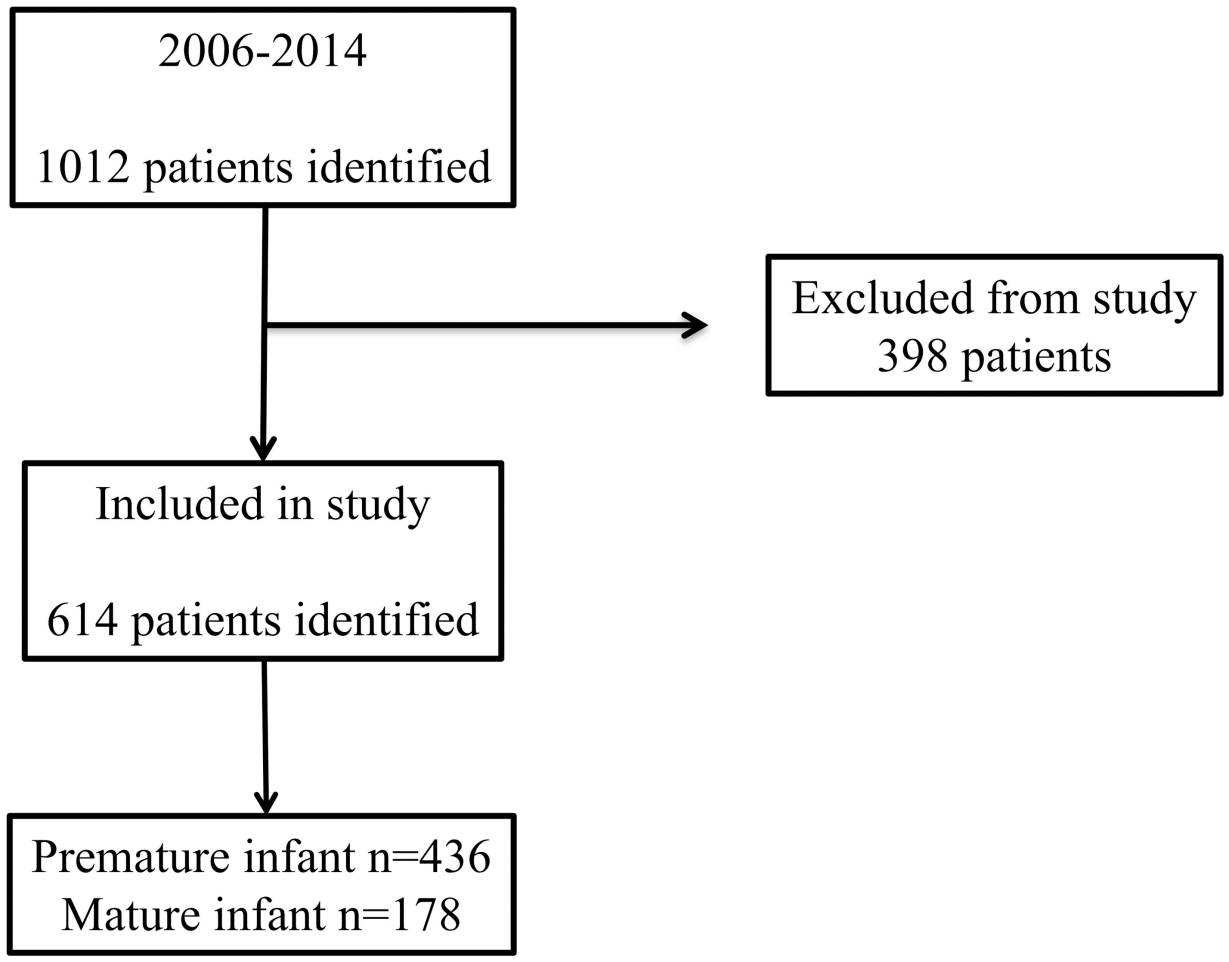
Flowchart of subjects in this study.

The flow chart in Fig 1 shows the number of patients available for recruitment and the actual number recruited. In this study of 1012 patients, 614 infants born at 22 to 41 wks were included. Of these patients, 436 and 178 were preterm and term infants, respectively. A total of 154 preterm infants born at <29 wks were admitted during the study period. The measured values of serum creatinine were shown in Tables 1 and 2. Table 1 shows the patients’ general characteristics. In infants born at <29 wks, median nSCr at birth and mSCr were 0.50mg/dl and 0.50mg/dl, respectively. However, in infants born at 29–36 wks and term infants, median nSCr at birth was significantly higher than mSCr, respectively (Table 1). The median nSCr at birth and mSCr in all groups were 0.56mg/dl and 0.50mg/dl, respectively (data not shown in Table 1). The median GAs at delivery were 31.9 wks and 38.0 wks in preterm and term infants, respectively. The median BWs were 1424g and 2736g in preterm and term infants, respectively. The median time of mother’s sampling is day0. And the median time of neonates sampling are day 7, day 14, and day 28, respectively.

**Table 1:** Characteristics of preterm and term infants enrolled in the study.

GA	< 29 wks	29−36 wks	>37 wks
N	154	282	178
nSCr at birth (mg/dl)	0.50	0.57*	0.60*
mSCr (mg/dl)	0.50	0.50	0.51
BW (g)	788	1423	2734
SGA (%)	23 (15)	59 (21)	21 (12)
RDS (%)	126 (82)	77 (27)	13 (7)
Antenatal Steroid (%)	67 (44)	81 (29)	16 (9)
Male (%)	78 (51)	136 (48)	96 (54)
Antibiotics (%)	77 (50)	79 (28)	57 (32)
Apgar Score (1min)	7	7	7
Apgar Score (5min)	8	8	8
INDO (%)	65 (42)	21 (7)	2 (1)

GA:gestational age, BW: birth weight, SGA: small for gestational age, RDS: respiratory distress syndrome, Antenatal steroid were bethamethasone, Antibiotics: the use of antibiotics to neonate, INDO: indomethacin administration to neonate. Continuous variables were presented as medians.

*:p<0.001 v.s. mSCr.

**Table 2:** Association of nSCr (mg/dl) and mSCr (mg/dl) at birth with characteristics of neonates and mothers using Mann—Whitney U test and Spearman’s rank correlation.

		<29 wks		29–36 wks		Preterm		Term	
		nSCr	mSCr	nSCr	mSCr	nSCr	mSCr	nSCr	mSCr
HELLP	Yes	0.48	0.50	0.54	0.53	0.57	0.50	0.56	0.43
	No	0.50	0.53	0.57	0.50	0.57	0.50	0.60	0.51
	p-value	0.798	0.243	0.743	0.506	0.943	0.928	0.534	0.083
PIH	Yes	0.66	0.52	0.59	0.52	0.61	0.52	0.69	0.53
	No	0.47	0.45	0.56	0.50	0.55	0.50	0.6	0.50
	p-value	**0.000**	**0.000**	0.141	**0.009**	**0.000**	**0.000**	**0.002**	0.609
Antenatal steroid	Yes	0.52	0.50	0.54	0.50	0.56	0.50	0.65	0.53
	No	0.55	0.49	0.58	0.50	0.58	0.50	0.6	0.50
	p-value	0.585	0.373	0.143	0.936	0.128	0.699	0.339	0.992
Mg	Yes	0.50	0.48	0.58	0.50	0.59	0.50	0.80	0.51
	No	0.50	0.50	0.56	0.50	0.56	0.50	0.60	0.50
	p-value	0.581	0.985	0.316	0.455	0.962	0.321	**0.004**	0.861
Male	Yes	0.51	0.48	0.56	0.50	0.56	0.50	0.60	0.50
	No	0.49	0.50	0.58	0.50	0.58	0.50	0.60	0.51
	p-value	0.282	0.704	0.24	0.977	0.716	0.754	0.256	0.629
RDS	Yes	0.52	0.50	0.58	0.50	0.53	0.50	0.7	0.50
	No	0.45	0.48	0.57	0.50	0.56	0.50	0.6	0.51
	p-value	0.389	0.916	0.81	0.053	0.046	0.642	**0.006**	0.953
SGA	Yes	0.70	0.54	0.64	0.53	0.66	0.54	0.62	0.5
	No	0.49	0.47	0.55	0.50	0.53	0.49	0.60	0.51
	p-value	**0.000**	**0.000**	**0.001**	**0.003**	**0.000**	**0.001**	0.544	0.942
BW	R	-0.20	-0.06	-0.17	-0.14	0.00	-0.01	-0.15	-0.08
	p-value	**0.012**	0.445	**0.016**	**0.017**	0.855	0.821	**0.038**	0.284
GA	r	0.24		0.04		0.16		-0.09	
	p-value	**0.002**		0.470		**0.001**		0.194	
Apgar Score (1min)	r	0.03	0.07	-0.01	-0.03	-0.03	0.00	-0.01	0.19
	p-value	0.668	0.384	0.686	0.554	0.425	0.941	0.850	**0.011**
Apgar Score (5min)	r	-0.02	0.04	0.02	-0.03	0.00	0.00	-0.06	0.18
	p-value	0.760	0.629	0.634	0.608	0.965	0.903	0.374	**0.014**
mSCr	r	0.76		0.71		0.74		0.34	
	p-value	**0.000**		**0.000**		**0.000**		**0.000**	

HELLP: hemolytic low platelet syndrome, PIH: pregnancy induced hypertension, Antenatal steroid were bethamethasone, Mg: magnesium administration to mother, RDS: respiratory distress syndrome, SGA: small for gestational age, INDO: indomethacin administration to neonate, Antibiotics: antibiotics administration to neonates, BW: birth weight, GA: gestational age, nSCr: neonatal serum creatinine levels, mSCr: maternal serum creatinine levels. Creatinine data were expressed as median. Continuous variables were analyzed using the Mann—Whitney U test. Significant correlation between the values of BW, GA, Apgar Score, mSCr, and nSCr were analyzed using Spearman’s rank correlation(r).

**Fig 2:**
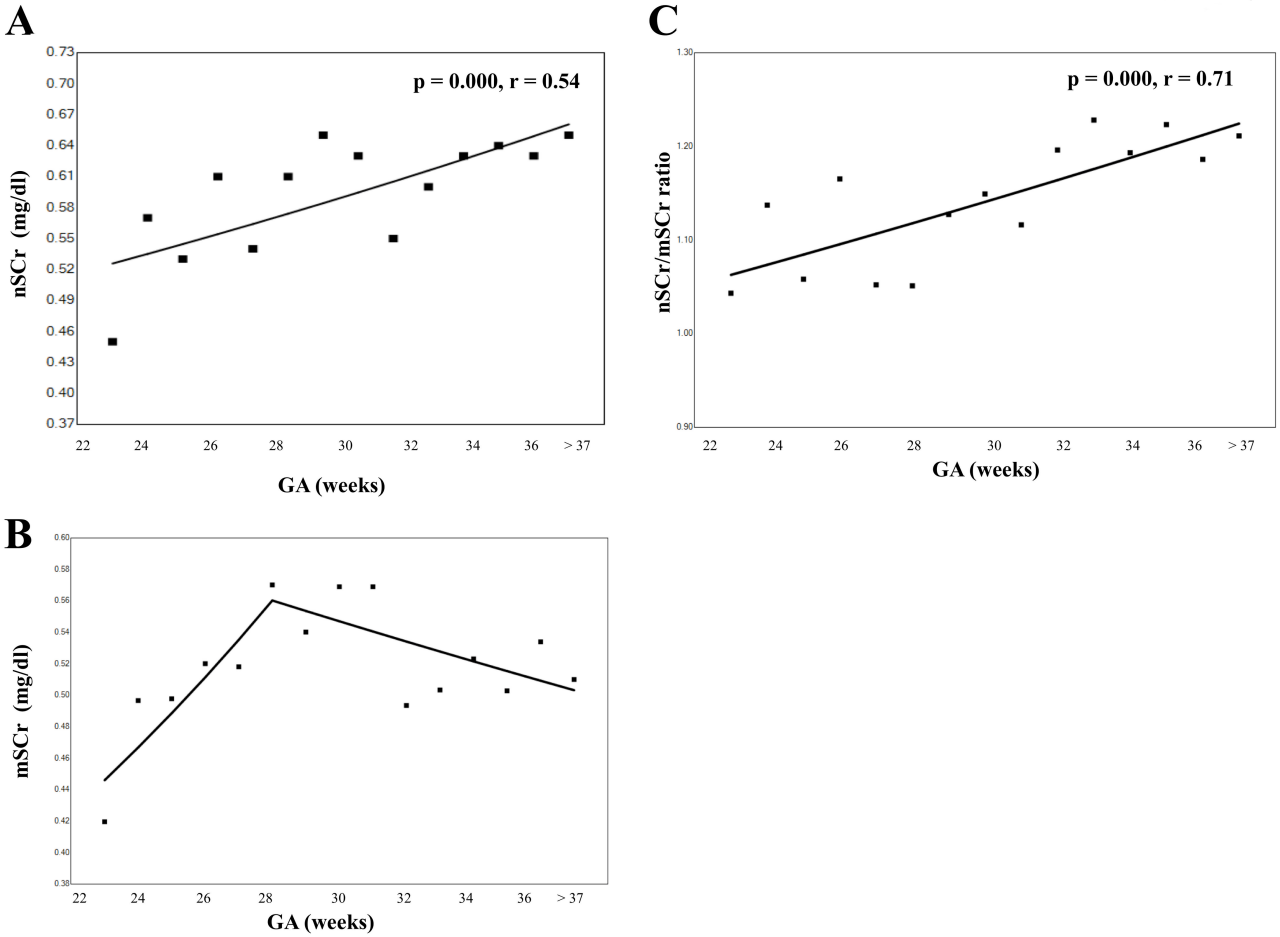
Correlation between nSCr, mSCr and GA. Joinpoint analysis using mean nSCr (A), mSCr (B), and ratio of nSCr/mSCr (C) from 22–41 weeks’ gestational age. Significant correlation between the values of GA, mSCr, and nSCr were analyzed using Spearman’s rank correlation(r) (A, C).

As shown in Fig 2, nSCr was significantly correlated with GA (p = 0.000, r = 0.54). However, mSCr had a joinpoint with 28 wks. mSCr at >28 wks gradually decreased with GA. Furthermore, nSCr/mSCr ratio was correlated with GA (p = 0.000, r = 0.71). Antenatal steroids, Apgar Score, maternal administration, gender and HELLP syndrome had no influence on nSCr at birth in preterm infants. As shown in Table 2, nSCr and mSCr with PIH were significantly higher than nSCr and mSCr without PIH in preterm delivery (p = 0.000). VIF values for all independent variables were less than 4.0. In neonates born at <29 wks, multivariate analysis showed that PIH (p = 0.000, β = 0.20) and mSCr (p = 0.000, β = 0.73) were significantly associated with nSCr. For infants born at 29–36 wks, multivariate analysis showed that only mSCr (p = 0.000, β = 0.70) was significantly associated with nSCr. In term infants, maternal magnesium administration (p = 0.000, β = 0.25), RDS (p = 0.013, β = 0.16), PIH (p = 0.005, β = 0.19), and mSCr (p = 0.000, β = 0.33) were significantly associated with nSCr (Table 3).

**Table 3:** Factors associated with nSCr and mSCr at birth using multivariate analysis.

		nSCr									
		Model 1						Model 2			
		<29 wks	29–36 wks	Preterm	Term			<29 wks	29–36 wks	Preterm	Term
BW	β	−0.81	−0.05	0.06	−0.1	GA	β	−0.04	0.03	0.1	−0.03
	p-value	0.096	0.274	**0.049**	0.119		p-value	0.356	0.416	**0.001**	0.632
SGA	β	0.04	0.04	0.11	**-**	SGA	β	0.08	0.07	0.08	**-**
	p-value	0.364	0.343	**0.000**			p-value	0.103	0.099	**0.006**	
Mg	β	**-**	**-**	**-**	0.25	Mg	β	**-**	**-**	**-**	0.24
	p-value				**0.000**		p-value				**0.001**
RDS	β	**-**	**-**	**-**	0.16	RDS	β	**-**	**-**	**-**	0.16
	p-value				**0.013**		p-value				**0.016**
PIH	β	0.20	0.00	0.07	0.19	PIH	β	0.21	0.00	0.07	0.20
	p-value	**0.000**	0.947	**0.025**	**0.005**		p-value	**0.000**	0.98	**0.015**	**0.002**
mSCr	β	0.73	0.70	0.71	0.33	mSCr	β	0.73	0.70	0.70	0.34
	p−value	**0.000**	**0.000**	**0.000**	**0.000**		p−value	**0.000**	**0.000**	**0.000**	**0.000**
		mSCr									
BW	β	0.05	-0.03	0.02	**-**	GA	β	0.20	0.01	0.07	**-**
	p-value	0.555	0.655	0.576			p-value	0.01	0.863	0.135	
SGA	β	0.10	0.09	0.11	**-**	SGA	β	0.05	0.11	0.10	**-**
	p-value	0.277	0.149	**0.016**			p-value	0.647	0.058	**0.033**	
PIH	β	0.26	0.19	0.21	**-**	PIH	β	0.26	0.2	0.22	**-**
	p−value	**0.003**	**0.001**	**0.000**			p−value	**0.003**	**0.001**	**0.000**	

BW: birth weight, GA: gestational age, SGA: small for gestational age, Mg: magnesium administration to mother, RDS: respiratory distress syndrome, PIH: pregnancy induced hypertension, nSCr: neonatal serum creatinine levels., mSCr: maternal serum creatinine levels. β means standardized regression coefficient.—: Variables with p-value > 0.05 at univariate analysis

In term infants, univariate analysis showed that only Apgar Score was significantly associated with mSCr (Table 2). In mSCr, multivariate analysis revealed that only PIH were significantly associated with mSCr in infants born at <29 wks (p = 0.003, β = 0.26) and 29–36 wks (p = 0.001, β = 0.19) (Table 3).

**Fig 3:**
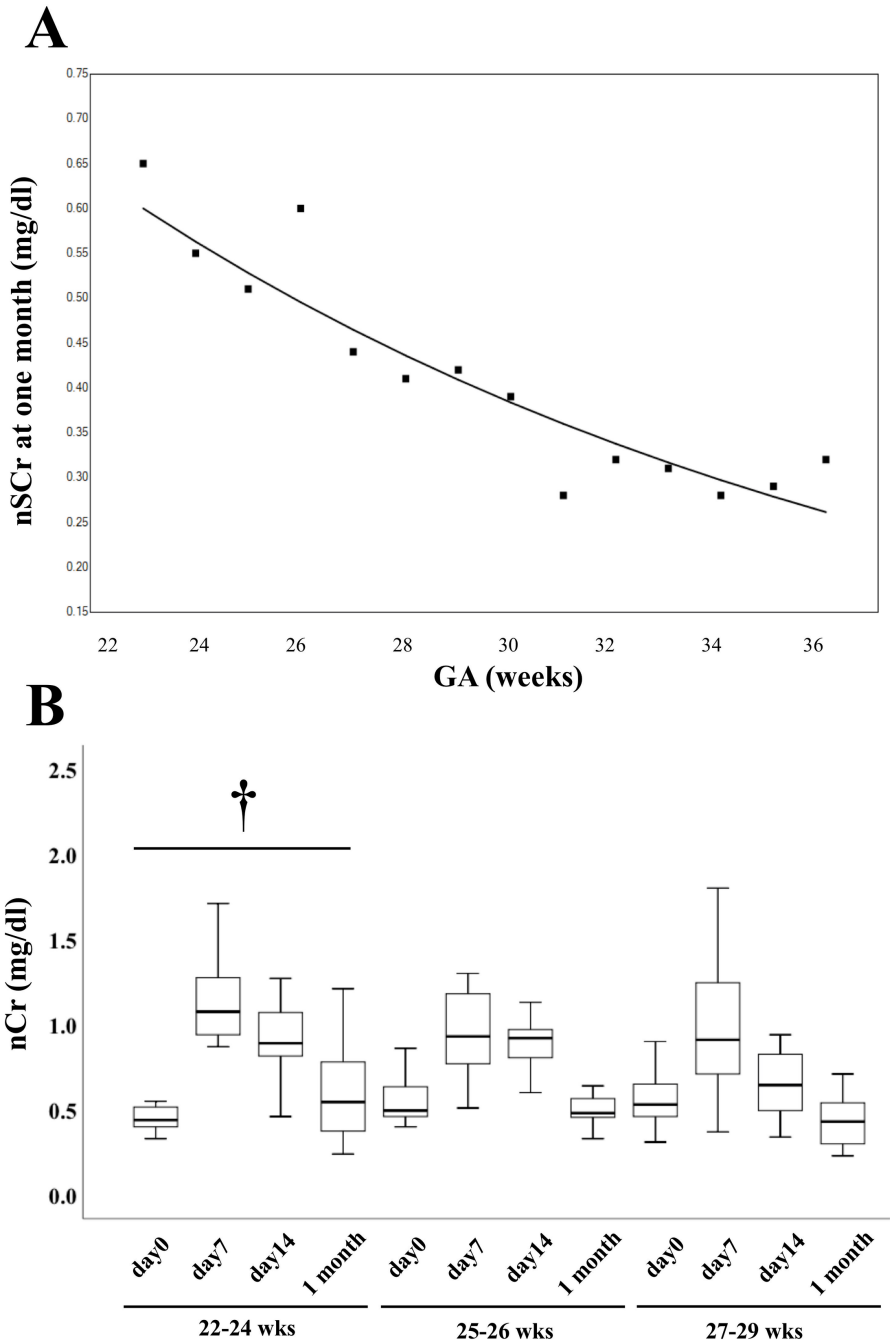
Creatinine levels during early postnatal period. A. nSCr of preterm infants born at <29 weeks’ gestational age from birth until four weeks of life. B. Serum and plasma creatinine levels in three groups of preterm infants born at gestationsal age of <29 weeks’ at one month are presented as box (interquartile range) and whisker (5%–95% range) plots. Creatinine at day 7 was plasma creatinine. †*P*<0.05.

In terms of nSCr at one month of life, univariate analysis showed that BW, GA, RDS, PIH, use of antibiotics, and use of indomethacin were significantly associated with nSCr in preterm infants (S1 Table). Multivariate analysis showed that GA (p = 0.007, β = −0.367) and PIH (p = 0.003, β = −0.171) were significantly associated with nSCr in preterm infants (Table 4, Fig 3A).

**Table 4:** Factors associated with neonatal creatinine levels at one month using multivariate analysis.

	<29 wks		29–36 wks		Preterm	
	(N = 138)		(N = 109)		(N = 247)	
	Β	P	β	p	Β	p
GA	−0.259	0.002	−0.209	0.028	−0.367	0.007
BW	-	-	-	-	−0.064	0.596
RDS	-	-	-	-	0.07	0.316
PIH	−0.235	0.004	-	-	−0.171	0.003
INDO	-	-	-	-	−0.025	0.69
Antibiotics	-	-	-	-	0.053	0.393
SGA	-	-	-	-	−0.092	0.2
mSCr	-	-	0.157	0.098	-	-

GA: gestational age, BW: birth weight, RDS: respiratory distress syndrome, PIH: pregnancy-induced hypertention, INDO: indomethacin administration to neonate, Antibiotics: antibiotics administration to neonates, SGA: small for gestational age, mSCr: maternal serum creatinine levels. β means standardized regression coefficient.

Furthermore, there was a significant difference in nSCr in infants born at <25 wks during the first month of life (Fig 3B). However, there was no differences in nSCr in infants born at ≧ 25wks.

## Discussion

Our study suggested that there was a relationship between nSCr and mSCr levels in Japanese neonates born with or without mother’s complications. Especially, nSCr and mSCr with PIH were significantly higher than nSCr and mSCr without PIH in preterm delivery. A previous study reported that fetal plasma creatinine levels reflected maternal creatinine levels from 15 wks until term [12]. Lao et al. reported that 84 full term neonates’ plasma creatinine levels at birth were completely the same as maternal creatinine levels [3]. On the other hand, Guignard reported a case born at <29 wks whose plasma creatinine levels were higher than that of the mother [12]. However, these studies had small sample sizes and did not reveal the relationship between nSCr and maternal conditions including mSCr from infants born at 22 wks to term. The present study was the first to report nSCr and mSCr levels at birth in a large cohort at different gestational ages and indicated that nSCr at birth in infants born at 29–36 wks and term infants was higher than mSCr as in a previous study [12]. In our study, the factors affecting nSCr in preterm infants was different from that of term infants. nSCr at birth in term infants was affected by maternal magnesium administration, PIH, and mSCr. Furthermore, our study also suggested that nSCr at birth correlates with GA as in a previous study [13]. Moreover, this study was the first report to find that there was a stronger correlation between nSCr and mSCr in preterm infants (p = 0.000, r = 0.74) compared with term infants (p = 0.000, r = 0.34).

In our study, the nSCr/mSCr ratio gradually increased with GA. This result was different from the previous study by Lao [3]. This difference may be due to the difference in the type of sample, the sample size, and GA. In Lao’s study, they studied full term infants and the sample size was 84. In our study, mSCr was not affected by GA, especially after 28 wks. This might have affected the nSCr/mSCr ratio after 28 wks. To date, there are no papers that report the correlation between mSCr and gestational age in Japanese population. Nduka et al reported that serum creatinine levels in Caucasians increased as pregnancy progressed, whereas that in Africans increased only slightly and only during the last trimester of pregnancy [14]. Conversely, Egwuatu showed that plasma creatinine in Nigerians increased during the second trimester, decreased in the third trimester [13]. In our study, mSC<28 wks increased and mSC>28 wks gradually decreased with gestational age as Egwuatu’s study.

Some studies have investigated creatinine levels in premature infants during the early postnatal period [15–22]. The present study indicated that nSCr at one month correlates with GA. Infants born at 22–24 wks GA are frequently exposed to potentially nephrotoxic medications and are more likely to have other risk factors for renal dysfunction. We have also shown that nSCr in premature infants born at 25–29 wks were elevated on the second week of life, and decreased postnatally to reach a plateau at one month. However, in infants born at 23–24 wks, nSCr were elevated on the first week of life, and nSCr did not reach a plateau at one month. Bateman et al. provided data showing that mean nSCr declined rapidly over three to seven weeks postnatally. In infants born at 25–27 wks, the decrease in mean nSCr begins midway through the first postnatal week and continues for six to seven weeks thereafter. In infants born at 28–29 wks, mean nSCr decreased from birth through six to seven weeks. Bateman et al. also reported that mean SCr from 10 to 14 days of life in neonates born at 25–27 wks and 28–29 wks were 66.3 umol/L and 61.0 umol/L, respectively [23]. In our study, nSCr levels at two weeks of life were almost the same as in the previous study. However, they did not investigate SGA infants and did not report nSCr levels at two weeks of life in neonates born at <24 wks. Thayyil et al. reported that the mean plasma creatinine level at two weeks of life in neonates born at <24 weeks was 110 umol/L [24]. In our study, the median nSCr in neonates born at <24 weeks was lower than in Thayyil’s study [24]. We speculate that these differences may have resulted from the way patients were managed in the NICU (intake on the first day of life, electrolyte levels, and ventilation). Initial intravenous fluid volume in our study was 70–90 mL/kg/day on the first day of life, progressively increasing to 100 mL/kg/day by the 72^nd^ hour of life.

Our study had several limitations. Although our study was one of the larger studies to explore nSCr in infants, it included a relatively small number of infants in the highest GA group. In this study, nSCr from early postnatal days should have been investigated, however, we did not collect samples from the earliest day of life to reduce phlebotomy losses in first weeks in our NICU policy. Gallini et al. reported that sCr peaked during the first days of life in preterm infants [25]. Auron and Mhanna et al. also reported that nSCr decreased significantly during the first days of life in VLBW infants. However, in infants born at <29 wks or those smaller than 1000 g BW, there was a delay in the decrease of nSCr that extended beyond the first days of life [21]. Furthermore, there was a lack of data about fluid intake, water balance, and hypernatremic dehydration during the first month of life, which might have affected nSCr levels. In the present study, we included the neonates (sCr>1.5mg/dl) because we speculated that mSCr correlates with nSCr. There are several reports suggesting that definition of acute kidney injury (AKI) is SCr> 1.5mg/dl [26,27]. The limitations of the AKI definition with serum creatinine have been discussed. Though there was a significant difference in nSCr at one month, this could be confounded by AKI especially in sick neonates. Recently a standardized classification of AKI has been proposed with RIFLE (risk, injury, failure, loss, end-stage) score in adults and pediatrics [28]. Ricci et al reported that RIFLE with a neonatal modification may lead to improvement in the knowledge of AKI incidence [29]. In this classification, the urine out put in the kidney injury is <1ml/kg/day. In this study, we adopted urine output criteria as inclusion criteria. Furthermore, we investigated creatinine levels of serum and plasma during postnatal period (Fig 3B). However, Rodney mentioned that there was no difference in creatinine levels between serum and plasma [30].

In summary, we conclude that nSCr reflected mSCr at all gestational ages, but the correlation between nSCr and mSCr in preterm infants was stronger than in term infants. We also conclude that the nSCr/mSCr ratio gradually increased with GA. However, in infants born at <29 wks, GA was a factor in the delayed decrease of their nSCr that extended beyond a month of life. In addition, nSCr at one month decreased with advancing GA.

## Supporting information

S1 Table Association of nSCr at one month with neonatal and maternal characteristics on univariate analysis. (DOC)
