# Supplementary material for: Neonatal and maternal serum creatinine levels during the early postnatal period in preterm and term infants
Source: PLoS One. 2018 May 24;13(5):e0196721. doi: 10.1371/journal.pone.0196721 (PMC5967735; doi:10.1371/journal.pone.0196721)
Supplement: S1 Table — (DOC) [file pone.0196721.s001.doc]

S1 Table Association of nSCr at one month with neonatal and maternal characteristics on univariate analysis.

|  |  | ＜29 wks |  |  | 29-36 wks |  |  | Preterm |  |
| --- | --- | --- | --- | --- | --- | --- | --- | --- | --- |
|  |  | nSCr at one month | p-value |  | nSCr at one month | p-value |  | nSCr at one month | p-value |
| HELLP | Yes  No | 0.57  0.50 | 0.544 |  | - | - |  | 0.50  0.39 | 0.151 |
| PIH | Yes  No | 0.43  0.50 | **0.004** |  | 0.30  0.30 | 0.183 |  | 0.33  0.40 | **0.015** |
| Male | Yes  No | 0.48  0.48 | 0.185 |  | 0.30  0.29 | 0.266 |  | 0.39  0.37 | 0.215 |
| Antenatal steroid | Yes  No | 0.50  0.46 | 0.212 |  | 0.30  0.28 | 0.896 |  | 0.40  0.39 | 0.191 |
| SGA | Yes  No | 0.35  0.50 | 0.001 |  | 0.30  0.29 | 0.160 |  | 0.30  0.40 | **0.001** |
| RDS | Yes  No | 0.48  0.46 | 0.164 |  | 0.30  0.29 | 0.228 |  | 0.45  0.30 | **0.000** |
| INDO | Yes  No | 0.49  0.46 | 0.548 |  | 0.34  0.29 | 0.334 |  | 0.47  0.35 | **0.000** |
| Antibiotics | Yes  No | 0.50  0.46 | 0.126 |  | 0.30  0.29 | 0.702 |  | 0.45  0.33 | **0.000** |

HELLP: hemolytic low platelet syndrome, PIH = pregnancy induced hypertension, Antenatal steroid were bethamethasone. SGA: small for gestational age, RDS: respiratory distress syndrome, INDO: indomethacin administration to neonate, Antibiotics: antibiotics administration to neonates, BW: birth weight, GA: gestational age, mSCr: maternal serum creatinine levels. Continuous variables were analyzed using the Mann- Whitney U test.
